# Book Review: Follow Your Gut: The Enormous Impact of Tiny Microbes

**DOI:** 10.3389/fmicb.2016.01312

**Published:** 2016-08-26

**Authors:** Daniel K. Morais, Júlia M. A. de Castro, Luiz F. W. Roesch, Victor S. Pylro

**Affiliations:** ^1^Biosystems Informatics and Genomics Group, René Rachou Research Centre – FIOCRUZBelo Horizonte, Brazil; ^2^Universidade Federal do PampaSão Gabriel, Brazil

**Keywords:** human microbiome, gut microbiome, human body, microbiology-science communication, microorganisms

The relationship between humans and microbes is attracting the scientific interest since the creation of the first lens by Leeuwenhoek (Fogg, 1969). As years went by, the understanding of the nature and importance of microbes became more apparent however, to date, for the broad audience, the understanding of the microbial roles in our life has been restricted to those causing diseases. Recent advances in the microbiome field, mostly boosted by the new DNA sequencing approaches, brought novel insights linking the human health and the microbes living in close contact with us (Loman and Pallen, 2015). Even though, a big effort from the non-scientific community to access these discoveries it is still necessary. The educational process has been undergoing remarkable changes in the forms of transmission and assimilation of knowledge. “Science”—as we know—is very recent in our history, being responsible for generating knowledge and technologies that profoundly impact the organization and the whole life of individuals. This book (Knight and Buhler, 2015) bridges the scientific knowledge and the general public with outstanding clarity. It uses an accessible language and everyday examples to explain complex concepts of microbial ecology and taxonomy. Moreover, it presents new discoveries regarding the role of microbes on diseases previously thought unrelated to them, like asthma, autism, and depression and links the gut microbes with our behavior.

Chapter 1 - This chapter shows how diverse microbes are and the metabolic importance of this diversity. It describes the microbial community present in different skin areas, in the nose and lungs, in the mouth and stomach, intestines, and genitals.

Chapter 2 - Describes how we acquire our first microbes from our mother and explains the agents influencing the microbial community shifts along our lives, in response to the food we eat, the contact we have with the environment and with pets, and the use of antibiotics. It ends discussing the links between microbiota diversity and health conditions, and the need for further studies in this area.

Chapter 3 - Links diseases like obesity, allergies, asthma, kwashiorkor malnutrition, and irritable bowel disease to the presence or absence of specific microbial groups and the life stages when we are exposed to them. The authors use several research papers showing evidences that the microbes have an enormous impact on our health.

Chapter 4 - Focuses on the influences that gut microbes have on our brain; it shows impacts on mood, behavior and/or psychological disorders. It presents several examples of experiments using mice with anxiety disorders or with induced autism, treated with probiotics and altering their gut microbiota, resulting in an alleviation of symptoms. Furthermore, the authors discuss the current state of art on the application of these treatment strategies to potentially cure human disorders. Experiments involving probiotics to help treating irritable bowel disease and celiac disease in infants are shown, and their relation to depression and several psychological effects are raised.

Chapter 5 - Starts driving the reader attention to the plasticity of our microbiome and raises the possibility of manipulating and enhancing it. To discuss this subject, the authors use a lawn as example, showing its whole biodiversity and how it grows. Later on, the authors introduce concepts of prebiotics—substances that enhance and benefit our microbes, and probiotics—microbes that live in and benefit the human body. Moreover, they talk about the risks of the microbe ingestion without the proper knowledge, discuss about the benefits brought by vaccines and consider a future, where we could use vaccines for diseases like depression, anxiety or other mind disorders.

Chapter 6 - Begins telling a story about the worries that people have when they think about vaccines and how comfortable they are about having antibiotics. This chapter explains the differences between these two issues, elucidating the effects on our whole microbiota and the risks of antibiotic misuse, like not having it for the corrected time, as prescribed.

Chapter 7 and Appendix - makes predictions about the future of the microbiome studies and the direct benefits it can bring to people. Furthermore, it introduces the three most famous American microbiome efforts—American Gut Project, Earth Microbiome Project and the Human Microbiome Project—and invites people to have their gut microbiomes described by the former.

## Author contributions

DM and JC read the book and write the review with contributions of LR and VP. All authors approved this work for publication.

## Funding

JC received studentship from Fundação de Amparo à Pesquisa do Estado de Minas Gerais (FAPEMIG) and Centro de Pesquisas René Rachou (CPqRR), through the PROVOC program.

### Conflict of interest statement

The authors declare that the research was conducted in the absence of any commercial or financial relationships that could be construed as a potential conflict of interest.
